# Uptake of home dialysis in younger adults: case studies that illustrate the multifaceted influence of home circumstances on dialysis decisions

**DOI:** 10.1002/ccr3.1254

**Published:** 2017-11-02

**Authors:** Louise Collingridge, Keri‐Lu Equinox, Serena Frasca, Rosemary Simmonds, Melinda Tomlins, Josephine Chow

**Affiliations:** ^1^ Honorary Associate, Department of Linguistics Faculty of Human Sciences Macquarie University North Ryde New South Wales Australia; ^2^ Cairns Hospital Cairns Queensland Australia; ^3^ Home Therapies Unit Renal Transplant Services Northern Adelaide Local Health Network Adelaide South Australia Australia; ^4^ Home Therapies Unit Barwon Health Geelong Victoria Australia; ^5^ Nephrology Department John Hunter Hospital Hunter New South Wales Australia; ^6^ South Sydney Western Area Health Service Liverpool Hospital Liverpool New South Wales Australia

**Keywords:** Home dialysis, nursing, nephrology, qualitative

## Abstract

Younger adults considering home dialysis need support to ensure home circumstances are suitable and affordable. Home circumstances relate closely to the financial burden reported by younger home dialysis users. Attention to home circumstances of younger patients with chronic kidney disease by policymakers, funders, and healthcare practitioners is needed.

## Introduction

Improved health, independence, flexibility, and the possibility of continuing or finding employment are all associated with home dialysis 1. Yet, fewer than 15% of hemodialysis patients and 25% of peritoneal or continuous ambulatory peritoneal dialysis patients take up the option of home treatment 2. Younger patients are commonly assumed to be well suited to home dialysis 3, although a recent meta‐analysis of qualitative studies identifying barriers and facilitators to home dialysis acknowledged a gap in knowledge regarding the home dialysis experience of younger adults 4.

Financial burden has been cited as a reason for the low uptake of home dialysis 5, 6. Financial burden is a complex notion, being relative to income and expenses, and one which is dependent on the broader healthcare context in any particular country. A significant cost for younger adults relates to homeownership and housing affordability, with younger adults experiencing the highest burden of homeownership costs 7. The in‐depth qualitative analysis in this study presents three dialysis cases, each with contrasting home circumstances. The first example is from a dialysis patient who has been renting a home and is transitioning to homeownership. The second case is an example of living in government‐assisted housing with dialysis. The third case is from someone who own built a home fit for home dialysis. These Australian cases offer insight into how homeownership is linked to financial burden. By highlighting home circumstances in relation to cost, this study highlights how living situation impacts on home dialysis decisions.

Exploring living circumstances has been largely unreported to date, but is important given that homes are the setting for treatment delivery in home dialysis 4. Understanding how home circumstances impact on home dialysis may be an important indicator as to the supports necessary to sustain home dialysis 8.

Individual narratives are retained to allow for understanding each of their unique experiences, allowing for sufficient context in each case to extract principles that might apply to other populations, even though local contexts (funding models, support, service delivery, and demographics) may be vastly different. These three case reports provide insights into healthcare practitioners as to how understanding home circumstances of younger adults with chronic kidney disease could support and sustain home dialysis.

## Methodology

The three cases reported here were collected as part of a multicentre, observational descriptive cohort study using mixed research methods, which has been fully explained in an earlier publication 9. The qualitative portion of the study followed guidelines contained within the Consolidated Criteria for Reporting Qualitative Research (COREQ) 10.

The first phase of the study required participants to complete a survey. Participants were recruited by site‐based researchers, all of whom were clinical staff associated with the participants. A total of 138 participants completed the first phase survey, and some volunteered to be interviewed in a follow‐up phase. Semistructured interviews were conducted with eleven participants. Each of the eleven interviews was conducted by one of two authors (LC or JC), neither of whom had a clinical relationship with any of the interviewees. LC is a qualitative researcher with an allied healthcare background, and JC is a specialist renal nurse and researcher. Three of the participants who were interviewed who were younger than the average age of participants (i.e., under 57 years) were selected for presentation in this study.

Semistructured interviews were conducted on hospital premises, in a quiet room, with the interviewer and interviewee present. In some cases, an additional member of the research team was present as an observer. Interviews were carried out according to a predesigned template. Interview topics were introduced under the following headings in order:

1. Experience (to date) of the healthcare system in relation to renal care
2. Identifying positive aspects of health care
3. Identifying what has not worked well and offering constructive criticism
4. Describing perceived burden and barriers to choosing home dialysis
5. Expenses incurred directly related to dialysis treatment over the previous 12 months
6. Identifying strategies which may facilitate choosing home dialysis.

Each interview lasted for approximately 30 min and was audio recorded. Interviewers recorded notes during each interview to contextualize ambiguities, if required. Interviews were conducted until data saturation was reached, as agreed between the interviewer and the observer from the research team.

The first author familiarized herself with all interviews by repeatedly listening to each recording. Written summaries of each case were then drawn up for each interview. Each interview was written up under headings linked to the questions asked by the interviewer. Each case retained its individual narrative 11, 12 but the summarized structure allowing for comparisons between how individual cases answered specific questions.

Individual summaries were read over by participating researchers at the sites where interviews were collected. No additional comments were made by researchers at any of the sites.

The primary researcher then identified themes, by coding accounts of the interviewees as recorded in the summaries. Themes were identified from the data, rather than being driven by a theoretical expectation 12. Common themes that emerged across the interviews are listed below in alphabetical order in Table 1.

**Table 1:** Themes and subthemes that emerged from interview data collected

Themes (in alphabetical order)	Multiple aspects of each theme
Age	Health and aging Age and treatment options Young age challenges Advanced age challenges
Control	Dialysis offering more control than waiting for disease to progress Homeownership offers control^a^ Control equated to normality
Costs	Rebates for utilities related to income and homeownership^a^ Savings versus costs Avoiding financial hardship with support Entitlements to hospital funding Aged care entitlements
Decision‐Making	Process, not one fixed decision Diagnosis and changing health needs Personal decision‐making Onset of dialysis timing Shifting between home and hospital Combining home and hospital treatments Training Evolving circumstances
Health System	Flexibility System Pharmacy supplies Comprehensive care Travel to appointments
Life events	Travel Homeownership^a^ Parenting Employment
Machine	Trust Systematic procedures
Parenting	Children accepting dialysis as normal Children as a resource Motivation and moral support
Social support	Family adjustment Carer support Mental health

a Indicates homeownership emerged under themes of control, costs, and life events.

The nine themes listed in Table 1 emerged across all six categories of questions. The multiple aspects of each theme also shown in Table 1 illustrate the multifaceted nature of each theme that emerged.

Provisional findings were presented to all on‐site researchers who were asked to comment on the clinical relevance of the findings, either in a group discussion or privately. On‐site researchers verified that the themes identified by the first author were clinically meaningful.

Three cases from adults, all younger than the average age of participants in the study (57 years) all illustrated complex relationships between themes. These three cases are presented as individual narratives below. These cases were selected because they illustrate the multifaceted and complex issues of homeownership (as related to income and employment). Within each case, quotations are provided that relate specifically to housing issues as they relate to their experiences of home dialysis. The cases are discussed to provide insight into how gaps in the health and social services could be filled.

Each case is presented as a narrative that retains the individual complexity to better understanding how themes combine to determine treatment decisions, which is in keeping with interpretive descriptions 11, 12 as well as suggestions by Morton and associates 13 for studies of decision‐making in dialysis patients. All cases have been deidentified for this report.

The study was authorized by local research governance bodies (ethics committees) at each participating site. Individual ethics approvals were received from the relevant committee from each site. All participants provided written consent to participate in the study.

## Case Reports

### Case 1 (Larry) – Transition from renting to homeownership

Larry (aged in his thirties) works full time in a scientific field in a tropical rainforest region of Australia. His partner has been caring for their two young children (both under 5 years of age) and is due to return to work shortly. Larry's sibling was diagnosed with kidney disease fifteen years ago, and Larry was approved as a donor for a kidney transplant that did not go ahead. No suggestion was made to Larry that he might have also been at risk for kidney disease at that time. Four years ago, Larry experienced symptoms that were ultimately linked to kidney disease of unknown origin. Initially, prescribed medication controlled Larry's kidney disease and he was placed on a waiting list for a renal transplant. With deterioration of his kidney disease and worsening of symptoms, Larry began dialysis approximately 12 months after diagnosis. Hemodialysis was chosen over peritoneal dialysis as it presented him with less risk of developing an infection from bacteria commonly found in his workplace than if he had undertaken peritoneal dialysis.

Home hemodialysis (HHD) was chosen for flexibility to both ensure ongoing employment for a secure income and to allow maximum time spent with his family. Larry has been on HHD for 3 years. He has the support of his partner and acceptance from his children. The time involved on dialysis (including machine cleaning, preparation, and dialysis itself) does interfere with Larry taking up other interests.

Larry reports that regular public hospital clinic visits are required to review his current health status, home dialysis treatment, and to review prescriptions for medication. However, he reports that the appointment times are not easily compatible with full‐time employment. The hospital issues letters with appointment times, which are usually not suitable for those in employment. In his experience, appointment schedules are rarely kept to by hospital staff, meaning that long‐waiting times at the hospital require Larry to take time off work, which amounts to a loss of income and disclosure of his medical condition at work – both of which he feels potentially place his employment at risk.

Return visits to hospital are required to renew prescriptions even though Larry's medical condition may be stable. Routine clinic appointments typically require Larry to repeat his medical history at each visit due to medical staff appearing to be unfamiliar with his case despite hospital records being available. Repeating his medical history each time both lengthens each visit and suggests to Larry that hospital staff members are not familiar with his circumstances. By contrast, staff members at the Home Training Unit are familiar with his circumstances and offer individualized support. He has access to emergency hospital staff by telephone and direct access to equipment suppliers – both of which have provided ongoing support for safe home dialysis.

When he started HHD, Larry was privately renting a house that had a room suitable for dialysis. Plumbing modifications were paid for by his health service. Where Larry lives, homeowners (not renters) are eligible for rebates for water and power (these utilities incur high ongoing costs for HHD patients). Securing and passing on rebates to the homeowner took several months. The house Larry was renting was subsequently put up for sale. Due to the insecurity that arose from renting, Larry and his partner decided to purchase a home rather than continue to rent. A major factor in deciding to secure their own home was the uncertain future for Larry, given his health risks, and the need for stable home circumstances to continue HHD. Larry reports that home dialysis enables him to keep working, so his employment is at risk when home dialysis is at risk. He also reports that work itself is tiring and that he is aware of his risk for stroke or other health complications due to added pressures at work.

Seeking to purchase a home proved more challenging than expected. A home loan was initially rejected by his bank, despite Larry's full‐time employment. Larry attributes rejection of his initial loan application to bank staff members being aware of his health condition and need for dialysis. A broker (not familiar with Larry's health history) was subsequently engaged to act as a third party, to secure a home loan and Larry and his family are due to move to their own home shortly.

The bank won't support us with that we even mentioned the home dialysis they still won't support us it's all there and they said I have to come back later on by that stage it's be too late and also the um the broker we are going through now doesn't know anything about my condition and we always make sure I have a long sleeve shirt silly as it sounds because we really have to secure this house (Larry)

Larry reports employment as the key to enable him to secure a home for himself and his family and to provide a base for him to dialyze at home, which can in turn ensure his ongoing employment. Larry sees homeownership and employment as both driving forces and necessary for home dialysis.

We had a lot of trouble because we don't actually own the house we don't pay the rates directly we obviously because we pay them through rent obviously but we don't don't do that no we don't have any sort of economic incentive other than the fact I can retain my position at work. It makes it really hard when you are working I know that I am probably quite a lot younger than a lot of people but I'm right in the middle of we're buying a house will be in 3 days’ time getting all finance sorted you know we've got other sort of impacts going on you know babies crying at night and stuff that maybe what some other patients might have so um you know it is important if I go to work that I get everything done as I need to.(Larry)

If I don't secure the house I don't secure my job if I don't secure my job I can't secure a mortgage if I can't secure a mortgage I can't secure a house it's a vicious circle so I guess that what I'm really sort of after now is that security you know is like I've got to have it though because I feel that I'm very sort of vulnerable at the moment with my whole family. (Larry)

#### Comment

Larry's account highlights that while younger dialysis patients may be assumed capable of carrying out home dialysis, ensuring the infrastructure of a home and ongoing employment to pay for the home may pose challenges that are not usually considered. Larry believed that disclosure of his need for dialysis prejudiced his first attempt to purchase a home even though that purchase would have secured his ability to continue dialysis at home, thereby relieving a burden on the health service. Younger adults who are dialysis patients may have to face obstacles like those faced by Larry, just to secure the infrastructure necessary to maintain dialysis at home. As few younger patients can be expected to own their own homes, those supporting people those who take on home dialysis ought to consider the housing situation and employment status and prospects of the individual in relation to their age, not offer advice based on age alone.

### Case 2 (Kate) – government‐assisted housing

Kate is a mother to five children aged between 17 years and 6 months. A visit to a hospital Emergency Department 5 years ago lead to a diagnosis of kidney disease. She understands that her kidney function deteriorated quickly (from 27% to 3% in 3 weeks). She reports that she started dialysis before fully recognizing the extent of her illness. Dialysis started in hospital with a temporary vascular catheter, and later, both a Tenckhoff catheter and an arteriovenous graft were inserted. Kate opted for home nocturnal Automated Peritoneal Dialysis (APD) until deteriorating health (pneumonia and raised potassium levels despite regular dialysis) forced a change to hospital hemodialysis, which lasted for 12 months, followed by a change to HHD.

PD gives you the freedom because you don't have to rely on anybody but yourself versus haemo. When I am at home I have a lot more freedom. (Kate)

After a short time on HHD, Kate discovered she was 21 weeks pregnant with her youngest child. During her pregnancy, Kate returned to hospital for dialysis 6 days per week. Her baby was born at 33 weeks and is now 6 months old. She is currently undergoing Facility hospital‐based hemodialysis, as she finds home hemodialysis is to be too difficult with such a young infant. Kate intends to return to HHD when able to cope with both her infant and home dialysis.

Kate reports that flexibility, time at home, and the convenience of not needing to find childcare are advantages of home dialysis. As a resident in public housing, Kate has received modifications to her home (paid for by the government) to allow for home dialysis. Modifications to public housing have extended to flooring and plumbing but not to air conditioning. Heat generated by her dialysis machine in an already warm room means that Kate feels that home dialysis during summer is neither comfortable nor safe. Investigations as to whether air conditioning could be installed are still underway. Without air conditioning for the room where she dialyzes, Kate suggests she would need to return to hospital‐based dialysis during hot weather. Such a decision has financial and social implications for her as she would need to find (and pay for) childcare.

Kate receives a disability support pension. She reports very poor insight into the demands of dialysis by the authorities she has consulted with. Kate was advised to seek full‐time work while undergoing dialysis and caring for her children.

They tried to tell me I could work 40 h per week while pregnant with bub and do this. I'm like are you an idiot? I spend 40 h a week sitting in a chair dialysing and I have children and I can't yeah it was all I could do to get them to approve my (disability pension) I was already on it they wanted to take me off it and a lot, I think a lot of the younger ones will find the same thing is happening now because if you are under 35 you shouldn't be on a disability pension because there is nothing wrong with you according to the government so it does make it very difficult for a lot of things. (Kate)

Kate estimates that home dialysis adds approximately $300 to her utility bills each month – before considering increased power demands with air conditioning – this sum is almost 20% of her disability support pension. Kate should be able to obtain rebates for utility costs (water and power) associated with dialysis. However, she reports that when she previously dialyzed at home, administrative delays and obstacles have meant that rebates were not readily applied, as they would have been on the basis that she is a disability support pensioner, not the recipient of an aged pension.

Most of us younger ones would be on a disability support pension – we don't get that (age pension rebates) so for us younger ones we just have to suffer the cost. (Kate)

Unless rebates and air conditioning can be secured, Kate feels she will need to continue to juggle the cost and inconvenience of childcare that would accompany continuing with hospital‐based dialysis against added utility bills.

We really require air conditioning…because otherwise I am stuck dialysing at midnight…60°C in my bedroom is not safe…in winter it is like having a heater but through summer it is not safe….they don't like to put heating or cooling in the houses. (Kate)

#### Comment

Younger patients report difficulty navigating support services. Kate's narrative illustrates that public funds to support home dialysis may be wasted without comprehensive attention to individual needs. Unless Kate can dialyze at home, which in her case would require air conditioning for a substantial portion of the year, the funding spent to modify her home would have been wasted. Costing of home dialysis may need to consider home modifications as well as other support services required to sustain home dialysis and make the investment worthwhile. Public education about kidney disease, for authorities responsible for approving rebates, would establish realistic employment expectations for dialysis patients with family responsibilities.

### Case 3 (Steve) – purpose‐built home for dialysis

Steve is qualified in both health care and the hospitality industry and was a high achiever in his chosen field until he was forced to stop work due to blood pressure problems associated with kidney disease 10 years ago. He had suffered severe internal injuries thirty years previously which he expected to lead to kidney failure at some point, as eventuated. Steve could plan for dialysis, understanding that he would require it at some point. He is financially independent due to careful planning and has appropriate private health insurance in addition to state‐funded hospital services. He prepared for a variety of treatment options, having both a Tenckhoff catheter for peritoneal dialysis inserted and an arteriovenous fistula created at the same time. He designed and built his own home, allowing for space and plumbing suitable for both types of home dialysis. His environmentally sustainable home relies on rainwater, which he reports may place him at risk for infection (although none has yet developed). Additionally, his house has solar power with a backup generator (which he feels is an advantage of self‐sufficiency).

Medical complications arose associated with Steve's Tenckhoff catheter insertion, which he reports were due to incompetence or negligence, but he nonetheless began peritoneal dialysis 3 years ago. He maintained work and a full family life until further medical complications occurred, which he attributes to PD. He had no alternative but to commence HHD until a surgical procedure was undertaken which allowed him to return to PD. PD remains his preferred method of dialysis. Although he currently dialyzes for 14 h of every 24 h, the effectiveness of PD is a current concern and he faces the prospect of changing back to HHD soon.

Steve reported that although he had anticipated starting dialysis 1 day and had prepared his home, finances, health insurances, and body, he was unprepared mentally for the impact of starting dialysis. He experienced a rapid change in health, which left him little time to accept and adjust to living with dialysis.

The changes in your life when you are diagnosed, not overnight, but within 2 weeks, are phenomenal. You go from being top of your professional field to being top of the waste pile, basically hugely damaging potentially. (Steve)

His home was prepared, and he fully anticipated needing dialysis, but he was nonetheless mentally unprepared to begin.

So I went from being feted to being an invalid. (Steve)

Steve's confidence in his consultant physician who attended to his health needs within his set of social circumstances assisted him to adjust to life with PD, his preferred treatment method. However, the health complications he experienced left no alternative but to undertake hemodialysis for an interval and he currently faces the prospect of returning to HHD. Steve reports that while he was prepared physically for HHD, he needed to address a needle phobia to be able to undertake HHD.

What I see is a massive hole in the system that does not necessarily pick up people who are struggling. (Steve)

He was not aware of support at this level from his Home Training Unit and so sought independent treatment from a healthcare practitioner.

I have a needle phobia, absolute needle phobia, which makes HHD a big challenge now you would think that the system would pick up on that on someone who is being trained in HD I've asked for help with that not three or four times for help. (Steve)

At the time of interview, he was facing the prospect of treating his needle phobia in preparation to a return to HHD on a more permanent basis.

That ties in with mental health, because if you can lead a relatively normal life um then you have a feeling of achievement and contribution and that makes a big difference to ones’ mental state. (Steve)

#### Comment

A long lead time and building a home designed for home dialysis did not prepare Steve for the quick deterioration in his health that took place, so that for him, the need for dialysis still took on the character of a rapid and sudden life‐changing event. He felt this most when he was forced to stop work due to health concerns, despite undergoing home dialysis, which he expected would help to keep him employed. Steve's case illustrated the need to prepare both mentally and physically for home dialysis and that even where home circumstances might be considered ideal and well prepared, comprehensive care involves considering mental and physical health.

## Discussion

Practitioners typically recommend home dialysis for younger patients 14, often assuming that home dialysis will enable them to take up employment, that in turn will allow for financial independence. The case reports above suggest a more complex relationship between income and age, as exemplified in homeownership, something which is typically less stable in younger patients.

Homeownership was a major factor in determining whether home dialysis could be sustained. Homeownership was different in each of the three cases reported, but in all three, homeownership was an important issue. Larry was seeking to purchase a home that provided stability for home dialysis. Steve had sufficient financial resources and time to plan for a home that could accommodate dialysis, and Kate was dependent on state provision of housing and home modifications to undertake home dialysis. Larry's concerns were to secure a base from which home dialysis could be maintained, which was a more primary goal than the home modifications offered to existing homes by hospitals. Even though having a home is fundamental to home dialysis, Larry believed he was discriminated against when applying for a home loan because of his need for dialysis. Despite being in full‐time employment, he needed to secure a broker who did not know of his condition, before a loan could be secured. Assisting dialysis patients to achieve homeownership or ensure very stable, long‐term rental arrangements may be a level of support that has been overlooked to date. Sauvé et al. 4 reported that home circumstances were not explored by the majority of qualitative studies included in their meta‐analysis of facilitators and barriers to home dialysis, which they found surprising. The cases presented here highlight the importance of housing to sustaining home dialysis. As younger adults are less likely to own their own homes, using Australia as an example 7, 15, home circumstances are less likely to be stable in younger dialysis patients, as exemplified in these cases, which suggest that support to ensure home circumstances are suitable for home dialysis would assist younger patients and may be a necessary step to supporting those on home dialysis who do not have stable home circumstances.

Increases in utility costs are understood as a reason the majority of patients choose dialysis in hospital, rather than home 16. Kate reported a high proportion of her monthly disability support income was needed to pay for utilities. Both Kate and Larry reported that some subsidies and rebates were available only to homeowners or those on aged pensions. Their reports are consistent with survey findings reporting that 84% of Australian dialysis patients aged 18–44 years reported financial strain, in spite of rebates being available 17.

Although home dialysis is thought to provide opportunities for employment, which in turn ought to fund homeownership, demands of employment for those needing dialysis is a complex issue. Steve and Larry are examples where ongoing employment or planning for stopping work after a successful career may mean less reliance on rebates to sustain home dialysis. As in the example of Steve, intentions to maintain work may not be fulfilled due to health concerns being affected by work. Work pressures as reported by Larry raise concerns about further deterioration in health. Kate's example shows family responsibilities to be added considerations that mean that home dialysis may not necessarily lead to possible employment. Steve and Larry both valued employment for money that could pay for buying or building a suitable home and paying ongoing bills. In their cases, employment made home dialysis possible. When Steve was forced to stop work, he reported an unwelcome change in role, in keeping with accounts that dialysis patients are less content when they are unemployed. The complex relationship between home dialysis and employment shown in these three cases illustrate that home dialysis will not necessarily lead to ongoing employment, and further, as shown in these case studies, a stable home circumstance, necessary to sustain home dialysis, could be at risk when employment is at risk.

All three cases show indications of adaptive coping. Larry did not disclose his medical condition, to securing a home loan, adapting to his circumstances. Steve designed a home, purpose built for dialysis. Kate sought rebates and facilities to accommodate home dialysis while caring for an infant.

These three narratives provide examples of what young dialysis patients need to do to sustain home dialysis. The summarized case reports retain the complexity of how commonly identified factors such as home circumstances interact with employment and support. Insights are provided for practitioners showing how individual experiences reflect multiple perspectives of a single phenomenon; in this case, how homeownership affects dialysis. While not generalizable to other dialysis patients, or broader populations, these case reports from Australia offer contrasting examples of what has happened in their individual cases. By describing the context of each case, they provide examples of how dialysis, a context‐sensitive treatment, can be experienced. Service providers and policymakers should caution against overly simplistic assumptions related to suitability for home dialysis based on individual factors such as age, financial factors, employment, or housing. Healthcare teams can incorporate discussions about social issues such as homeownership, as they seek to address the widely reported decreasing number of patients taking up home dialysis across the world.

## Authorship

LC: conducted interviews, prepared data for analysis, coordinated the qualitative analysis, and prepared the manuscript as the associate researcher for this project. K‐LE, SF, RS, and MT: facilitated data collection and provided input to the analysis and the final manuscript as site co‐ordinators. JC: conducted interviews, identified major themes for analysis, and contributed to the discussion of cases as the senior researcher.

## Conflict of Interests

Josephine Chow, Melinda Tomlins, Keri‐Lu Equinox, Rosemary Simmonds, and Serena Frasca are members of The HOME Network, a group of professionals who promote home dialysis in Australia.
